# Platelet Adhesion Mediated by von Willebrand Factor at High Shear Rates Is Associated with Premature Coronary Artery Disease

**DOI:** 10.3390/biomedicines11071916

**Published:** 2023-07-06

**Authors:** Sergey Okhota, Sergey Kozlov, Yuliya Avtaeva, Ivan Melnikov, Olga Saburova, Konstantin Guria, Evgeny Matroze, Zufar Gabbasov

**Affiliations:** 1 Department of Problems of Atherosclerosis, National Medical Research Centre of Cardiology Named after Academician E.I. Chazov of the Ministry of Health of the Russian Federation, 121552 Moscow, Russia; bestofall@inbox.ru; 2 Laboratory of Cell Hemostasis, National Medical Research Centre of Cardiology Named after Academician E.I. Chazov of the Ministry of Health of the Russian Federation, 121552 Moscow, Russia; julia_94fs@mail.ru (Y.A.); ivsgml@gmail.com (I.M.); saburovaos@mail.ru (O.S.); kgguria@gmail.com (K.G.); matroze.eg@phystech.edu (E.M.); 3 Laboratory of Gas Exchange, Biomechanics and Barophysiology, State Scientific Center of the Russian Federation—The Institute of Biomedical Problems of the Russian Academy of Sciences, 123007 Moscow, Russia; 4 Department of Innovative Pharmacy, Medical Devices and Biotechnology, Moscow Institute of Physics and Technology, 141701 Dolgoprudny, Russia

**Keywords:** von Willebrand factor, GPIb, platelet adhesion, coronary artery disease

## Abstract

This study investigated von Willebrand factor (VWF)-mediated platelet adhesion at high shear rates in patients with premature coronary artery disease (CAD). The study included 84 patients with stable premature CAD and 64 patients without CAD. Whole blood samples were perfused through a microfluidic cell over a collagen-coated surface at a shear rate of 1300 s^−1^. Measurements were performed before and after the inhibition of VWF-specific platelet GPIb receptors with an anti-GPIb monoclonal antibody (mAb). Platelet adhesion decreased by 77.0% (55.9; 84.7) in patients with premature CAD and by 29.6% (0.0; 59.7) in control patients after the inhibition of VWF–platelet interaction with anti-GPIb mAb (*p* < 0.001). After adjusting for traditional risk factors, the odds ratio for premature CAD per 1% decrease in GPIb-mediated platelet adhesion was 1.03 (95% CI, 1.02–1.05; *p* < 0.001). The optimal cut-off level value of GPIb-mediated platelet adhesion was 62.8%, with 70.2% sensitivity and 81.2% specificity for CAD. The plasma levels of VWF or antiplatelet therapy did not affect the GPIb-mediated component of platelet adhesion. Thus, the GPIb-mediated component of platelet adhesion was more pronounced in patients with premature CAD. This may indicate the possible role of excessive VWF–platelet interactions in the development of premature CAD.

## 1. Introduction

Premature coronary artery disease (CAD) is CAD that begins in men before the age of 55 and in women before the age of 65 [1]. The state of the hemostasis system and, particularly, an increased tendency to thrombosis may be among the factors contributing to the early onset of CAD. Thrombosis occurs under conditions of high blood flow velocities in the arterial bed. Blood is a heterogeneous fluid, and its layers move at different velocities. The velocity at which some fluid layers move relative to others is called the shear rate; it is expressed in reciprocal seconds (s^−1^) [2]. A higher flow velocity and a narrower arterial lumen are associated with a higher shear rate.

Exposure to high shear rates prevents the direct binding of platelets to the damaged arterial wall [2]. The involvement of von Willebrand factor (VWF) is necessary for platelet adhesion under such conditions [2,3]. It is a large multimeric protein mainly contained in blood plasma and, in smaller amounts, platelet α-granules. VWF is sensitive to shear rate. VWF multimers are activated at shear rates characteristic of stenotic arteries [2,3]. Activated VWF binds to the collagen of the damaged arterial wall and facilitates platelet adhesion [2,3].

Taking into account the fact that premature CAD frequently begins with acute atherothrombotic events [4] and often leads to adverse outcomes [5], we hypothesize that such events might be related to the features of thrombus formation at the initial stage, where VWF plays a key role. The aim of this study was to investigate platelet adhesion to collagen mediated by the interaction between platelet glycoprotein Ib (GPIb) receptors and VWF in patients with premature CAD. GPIb is the only receptor on inactivated platelets specific to VWF [6]. This study was performed using an original microfluidic device [7], which allows for the simulation of the blood flow at a shear rate characteristic of a stenotic coronary artery segment.

## 2. Materials and Methods

### 2.1. Study Design

The study included 84 patients with stable CAD, including 62 men under 55 years of age with CAD onset before 50 years of age and 22 women under 65 years of age with CAD onset before 60 years of age. These patients underwent coronary angiography (CAG), which revealed stenotic lesions in the coronary arteries. The control group included 64 patients (26 men under 55 years of age and 38 women under 65 years of age) without clinical manifestations of CAD. They had no stenotic coronary atherosclerosis detected via CAG and/or computed tomographic angiography of the coronary arteries. A stenotic lesion was considered a lesion resulting in a 50% or greater reduction in the lumen of the left main, left anterior descending, left circumflex, and right coronary arteries, or a second-order branch with a diameter >2 mm [8].

The study did not include patients with familial hypercholesterolemia; low-density lipoprotein (LDL) cholesterol >4.9 mmol/L; unstable angina, in the first 2 months after myocardial infarction (MI), bypass grafting or coronary angioplasty; with NYHA functional class III–IV heart failure; left ventricular ejection fraction <40%; a permanent form of atrial fibrillation/flutter; aortic or mitral stenosis; hereditary and acquired coagulopathies; malignant neoplasms; and clinical and laboratory signs of acute infectious disease within the previous two months.

The presence of traditional risk factors for CAD (male sex, age, family history of CAD, high-density lipoprotein (HDL) cholesterol level <1.0 mmol/L for men and <1.2 mmol/L for women, LDL level >3 mmol/L, smoking, obesity, diabetes mellitus, and arterial hypertension) was assessed in all patients and compared between the two study groups.

### 2.2. Measurement of VWF Plasma Levels

VWF plasma (VWF:Ag) levels in patients were measured using an enzyme-linked immunosorbent assay (ELISA) (Thermo Fischer, Waltham, MA, USA). The units of VWF plasma concentration were taken as the percentage of normal content (50–150%).

### 2.3. Blood Sample Collection

Blood samples were collected from the cubital vein in S-Monovette 3.8% sodium citrate vials and S-Monovette vials (Sarstedt, Nümbrecht, Germany) with 100 µmol D-phenylalanyl-L-propyl-L-arginine-chloromethyl ketone (PPACK) (Enzo, Farmingdale, NY, USA). PPACK-anticoagulated blood was used to perform platelet adhesion measurements. All measurements in whole blood were performed 2 h after sample collection. Platelet-poor plasma was prepared through the centrifugation of citrated blood samples for 20 min at 2000× *g* and stored at −70 °C. The plasma was thawed in a water bath at 37 °C before measurements were taken.

### 2.4. Materials for Platelet Adhesion Measurements

The surfaces of glass slides were coated with type I rat collagen (Sigma-Aldrich, St. Louis, MO, USA) via incubation with a 0.1 mg/mL collagen solution in phosphate-buffered saline (Sigma-Aldrich, St. Louis, MO, USA) for 2 h at room temperature. Prior to coating, the slides were rinsed with 70% ethanol and dried. The monoclonal antibody (mAb) AK2 against the ligand-binding domain GPIb of the platelet membrane GPIb-IX complex was a gift from Dr. A. Mazurov, from the National Medical Research Center of Cardiology. The collagen solution was stored at 4 °C, while the mAb was stored at −70 °C.

### 2.5. Measurement of Platelet Adhesion to the Collagen Surface

**Figure 1:**
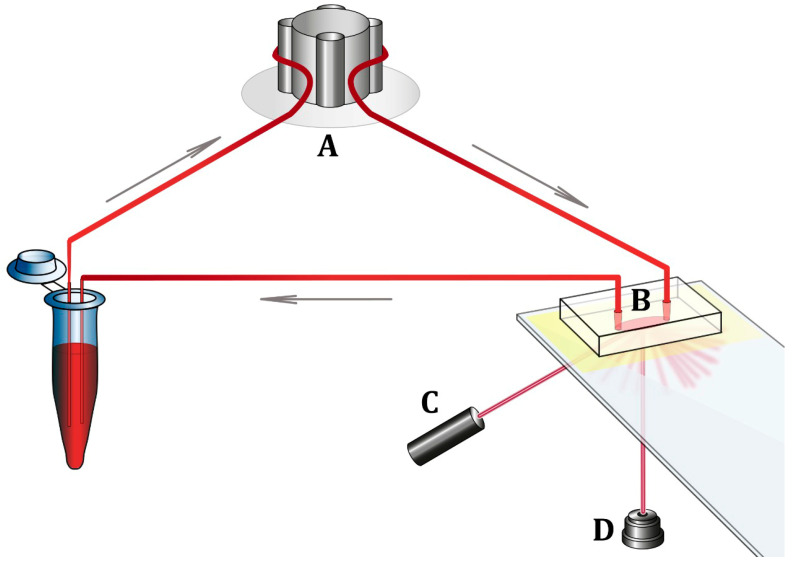
Microfluidic device for recording platelet adhesion kinetics under controlled flow conditions. (**A**) Peristaltic pump; (**B**) flow chamber; (**C**) semiconductor laser with emission wavelength λ = 650 nm; (**D**) photodetector of scattered laser radiation.

A microfluidic device for recording the kinetics of blood cell adhesion to the protein-coated surface under controlled flow conditions was developed at the Chazov National Medical Research Centre of Cardiology [7]. The device consists of a flow chamber with a collagen-coated optical surface, a peristaltic pump ensuring blood movement through the flow chamber, a laser, a photodetector, and an analog-to-digital converter connected to a computer (Figure 1). In the first stage of the experiment, whole PPACK-anticoagulated blood was placed in a microtube and connected to the system, ensuring blood flow in the flow chamber. Laser radiation was directed to the collagen-coated optical surface of the flow chamber. Scattered laser radiation was captured by a photodetector. When the device was switched on, whole blood moved inside the flow chamber at a preset speed. The shear rate was ≈1300 s^−1^, which is considered characteristic of arteries with moderate lumen stenosis [9,10]. Blood cells and, primarily platelets, interacted with the collagen coating and adhered to it inside the flow chamber. This caused the scattering of laser radiation, which increased as the number of adherent cells on the collagen-coated surface increased. The scattered laser radiation recorded by the photodetector was converted into electric voltage and measured in millivolts (mV). Thus, the increase in electric voltage at the photodetector output corresponded to the increase in the extent of cell adhesion to the collagen-coated surface. The circulation of blood in the system and recording of the photodetector signal lasted for 15 min. The extent of platelet adhesion was determined by the final value of the photodetector signal at the end of the 15-min blood circulation. Changes in scattered light intensity registered by the photodetector were recorded using the software L-Graph2 version 2.35.16 (L-CARD, Moscow, Russia). In the second stage of the experiment, 10 µg of mAb AK2 against GPIb receptors was added to a new whole blood sample, and 15-min blood circulation through a new flow chamber was repeated. The results of the measurements were compared between groups of patients.

### 2.6. Statistical Analysis

Quantitative data collected in the study are presented as median and quartile (lower quartile, upper quartile). The Shapiro–Wilk W-test was used to test statistical hypotheses regarding the distribution type. Different methods of nonparametric statistics were used for the comparative analysis of patient data in both groups: Fisher’s exact test and the χ^2^ test with Yates’s correction when comparing qualitative characteristics; the Mann–Whitney U-test when comparing quantitative characteristics in two independent groups; the Kruskal–Wallis test when comparing quantitative characteristics in three or more independent groups; the Wilcoxon test when comparing quantitative characteristics in two dependent groups; and the Friedman test when comparing quantitative characteristics in three or more dependent groups. Spearman’s rank correlation was used to assess the correlation between VWF:Ag levels and platelet adhesion. The association between a relative decrease in platelet adhesion after GPIb inhibition and premature CAD was assessed using logistic regression analysis and expressed as an odds ratio (95% confidence interval (CI)). The *p*-value was significant at 0.05. All tests were two-tailed. Statistical analysis was performed using Statistica v. 7.0 (StatSoft Inc., Tulsa, OK, USA) and SPSS Statistics v. 26.0 (SPSS Inc., Chicago, IL, USA) software.

### 2.7. Ethical Approval

The study followed good clinical practice standards and the Declaration of Helsinki principles. This study was approved by the Ethics Committee of the Chazov National Medical Research Centre of Cardiology of the Ministry of Health of the Russian Federation, Moscow, Russia (minutes No. 262, 30 November 2020). Written informed consent was obtained from all participants.

## 3. Results

Patients with premature CAD were more commonly male, had diabetes mellitus, HDL cholesterol <1.0 mmol/L for men and <1.2 mmol/L for women, LDL cholesterol >3 mmol/L, were more often smokers and heavy smokers, and had a higher smoking index value (Table 1). MI, as the first manifestation of CAD, was equally common in both men and women and was registered in 42.8% of cases. Among patients with CAD, 79.7% underwent coronary artery stenting, and 9.5% underwent coronary artery bypass grafting. Stenotic lesions of the left main coronary artery were detected in 11.2% of cases, the left anterior descending artery in 79.7%, the left circumflex artery in 56.2%, and the right coronary artery in 73.7% of cases.

In patients with premature CAD, the median VWF:Ag level was 111% (85; 143), whereas in the control group it was 145% (99; 189), *p* = 0.03. VWF:Ag levels were independent of age, sex, family history of CAD, smoking, obesity, diabetes mellitus, HDL, and LDL cholesterol levels. VWF:Ag levels were higher in patients with arterial hypertension compared with patients without it: 129% (97; 183) and 110% (73; 133), respectively, *p* = 0.04.

**Figure 2:**
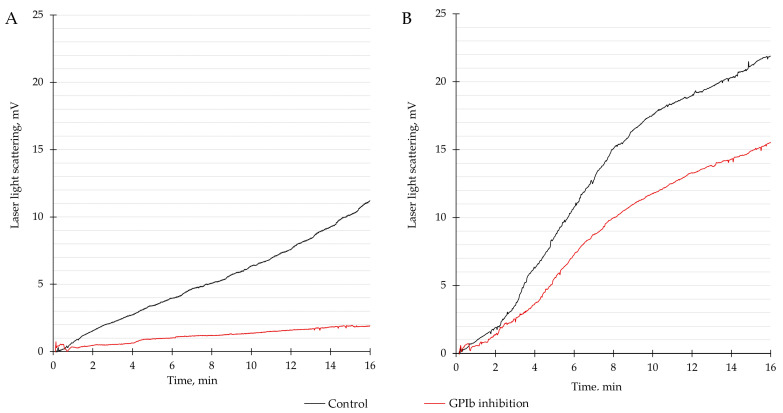
Typical curves of the time-dependent increase in the intensity of the scattered laser light recorded during the 15-min circulation of whole blood samples. Control measurements (black) and measurements with glycoprotein Ib (GPIb) inhibition with a monoclonal antibody (red) are shown for (**A**) a patient with premature CAD and (**B**) a control patient without CAD. The measurements were performed in a microfluidic device with a flow chamber that had a glass bottom coated with type I collagen. The shear rate in the flow chamber was 1300 s^−1^. The platelets interacted with and adhered to the collagen-coated surface. This caused the scattering of the laser light, which had been increasing as more platelets adhered to the surface. Scattered laser light was registered by the photodetector and converted into voltage measured in millivolts (mV). An increase in voltage at the output of the photodetector corresponded to an increase in the extent of platelet adhesion to the collagen-coated surface.

In patients with premature CAD (Figure 2A), the median 15-min platelet adhesion value was 8.3 mV (5.2; 13.5) at baseline and 1.7 mV (1.3; 3.3) after GPIb inhibition, which constituted a 77.0% (55.9; 84.7) relative decrease (*p* < 0.001). In control patients (Figure 2B), the median 15-min platelet adhesion value was 12.6 mV (9.2; 17.0) at baseline and 10.3 mV (4.3; 14.9) after GPIb inhibition, which constituted a 29.6% (0.0; 59.7) relative decrease (*p* = 0.001). The inhibition of GPIb resulted in a greater reduction in platelet adhesion in patients with premature CAD compared with the control group (*p* < 0.001) (Table 2).

**Figure 3:**
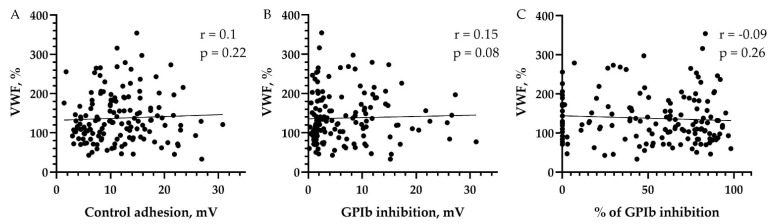
Correlation of VWF:Ag levels with (**A**) baseline values of platelet adhesion; (**B**) values of platelet adhesion after GPIb inhibition; and (**C**) values of a relative decrease in platelet adhesion after GPIb inhibition. VWF—von Willebrand factor; mV—millivolt; GPIb—glycoprotein Ib. Correlation analysis was performed using Spearman’s rank correlation coefficient.

VWF:Ag levels did not correlate with baseline platelet adhesion values, platelet adhesion values after GPIb inhibition, or the values of a relative decrease in platelet adhesion after GPIb inhibition in the entire cohort (Figure 3).

**Figure 4:**
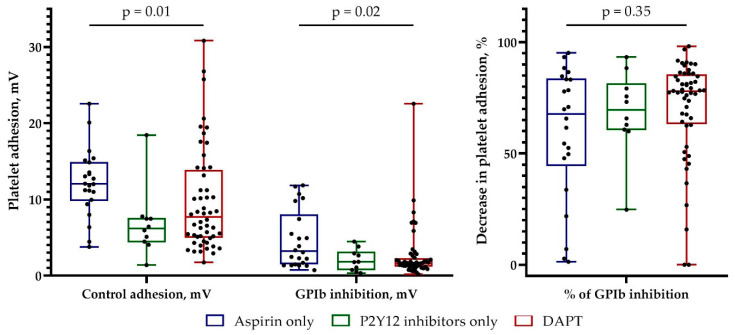
The effect of antiplatelet therapy on platelet adhesion in patients with premature coronary artery disease. mV, millivolt; GPIb, glycoprotein Ib; DAPT, dual antiplatelet therapy; *p*, comparison of three or more independent groups (Kruskal–Wallis test).

Among patients with premature CAD, 22 patients received only aspirin, 10 patients received only P2Y_12_ receptor inhibitors, and 52 patients received dual antiplatelet therapy (DAPT). The absolute values of platelet adhesion were less pronounced in patients receiving P2Y_12_ inhibitors or DAPT compared with patients receiving only aspirin. Nonetheless, a relative decrease in platelet adhesion after GPIb inhibition did not differ depending on the antiplatelet therapy used (Figure 4).

**Figure 5:**
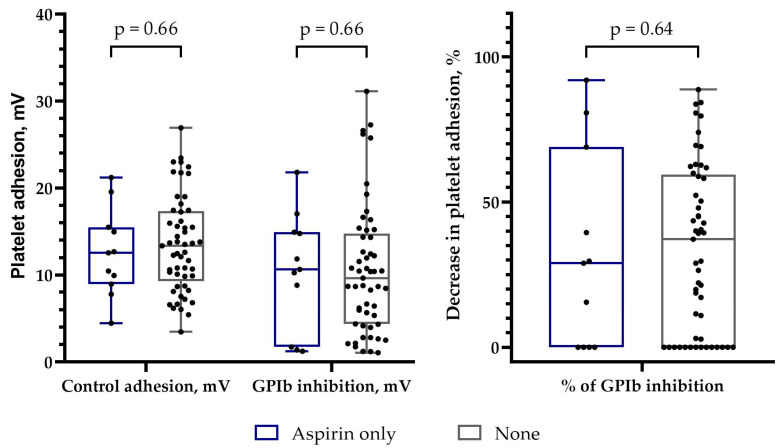
The effect of antiplatelet therapy on platelet adhesion in control group patients. mV—millivolt; GPIb—glycoprotein Ib; DAPT—dual antiplatelet therapy; *p*—comparison between two independent groups (Mann–Whitney U test).

In the control group, 11 patients received only aspirin, whereas 53 patients did not receive any antiplatelet therapy. Aspirin therapy did not affect the absolute values of platelet adhesion or the values of a relative decrease in platelet adhesion after GPIb inhibition (Figure 5).

**Figure 6:**
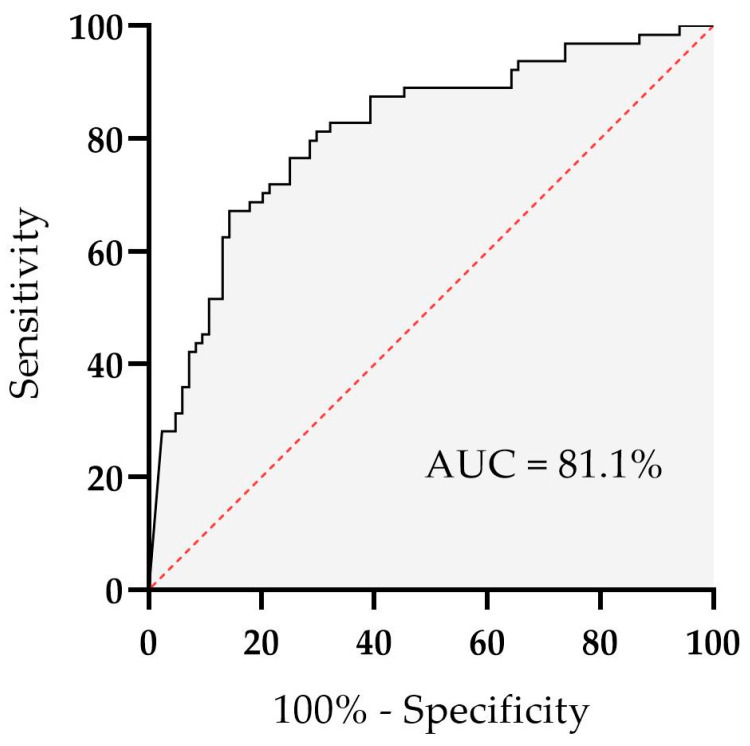
Receiver operating characteristic (ROC) analysis for a relative decrease in platelet adhesion after GPIb inhibition and its association with premature coronary artery disease. The area under the curve (AUC) was 81.1% ± 3,6% (95% confidence interval (CI) 73.9–88.2%, *p* < 0.001). GPIb, glycoprotein Ib. An optimal cut-off threshold value for a relative decrease in platelet adhesion after GPIb inhibition was 62.8%, with 70.2% sensitivity and 81.2% specificity for premature CAD.

A receiver operating characteristic (ROC) analysis was performed to assess the association of values of a relative decrease in platelet adhesion after GPIb inhibition with premature CAD (Figure 6). The area under the curve (AUC) was 81.1% ± 3.6% (95% CI, 73.9–88.2%, *p* < 0.001). The best combination of sensitivity (70.2%) and specificity (81.2%) was achieved at a cut-off threshold value of 62.8% of a relative decrease in platelet adhesion after GPIb inhibition. A relative decrease in platelet adhesion after GPIb inhibition was equal to or higher than the cut-off value in 59 (70.2%) patients with premature CAD and in 12 (18.8%) control patients.

In a univariate logistic regression analysis, a relative decrease in platelet adhesion after GPIb inhibition, male sex, diabetes mellitus, HDL cholesterol values <1.0 mmol/L for men and <1.2 mmol/L for women, smoking, and age were independently associated with premature CAD (Table 3).

The unadjusted odds ratio for premature CAD per 1% decrease in GPIb-mediated platelet adhesion was 1.04 (95% CI, 1.03–1.06; *p* < 0.001). This odds ratio was adjusted for other risk factors and biomarkers through multivariate logistic regression analysis. The logistic regression model is characterized in detail in the Supplementary Materials (Tables S1–S4). The model included a relative decrease in platelet adhesion after GPIb inhibition, male sex, smoking, and age. Other risk factors and biomarkers were excluded because they decreased the predictive value of the model. The model was significant (*p* < 0.001), with 81.1% of overall correct predictions for premature CAD. The adjusted odds ratio for premature CAD per 1% decrease in GPIb-mediated platelet adhesion was 1.03 (95% CI, 1.02–1.05; *p* < 0.001) (Table 4).

## 4. Discussion

The proposed hypothesis was confirmed by the results of this study. VWF in circulating blood interacted with the collagen-coated surface of the flow chamber and facilitated platelet adhesion. This led to an increase in the intensity of laser radiation scattered from the optical surface of the chamber. The addition of mAb to platelet GPIb receptors in circulating blood samples resulted in a reduction in adhesion and a decrease in laser light scattering. A decrease in platelet adhesion after GPIb inhibition was more pronounced in patients with premature CAD than in the control group. Patients with premature CAD showed a 77.0% relative decrease in platelet adhesion after GPIb inhibition, whereas for patients in the control group, this value was only 29.6% (*p* < 0.001). Therefore, it can be assumed that the contribution of the VWF–platelet interaction through GPIb receptors to platelet adhesion in patients with premature CAD is higher than in patients without CAD. The logistic regression analysis showed that the adjusted odds ratio for premature CAD per 1% decrease in GPIb-mediated platelet adhesion was 1.03 (95% CI, 1.02–1.05; *p* < 0.001).

In this study, the values of baseline adhesion measurements were lower in patients with CAD than in control subjects. Treatment with aspirin alone did not affect platelet adhesion in CAD or in the control group. Unlike control subjects, most patients with CAD received a P2Y_12_ receptor inhibitor alone, or DAPT. It was previously shown that aspirin did not decrease platelet aggregation and thrombus formation at high shear rates, possibly due to the suppression of prostacyclin (prostaglandin I2) production [6]. Prostacyclin is a potent inhibitor of platelet adhesion and aggregation, especially at high shear rates [6]. P2Y_12_ inhibitors downregulate the activation of platelet integrins through a different pathway and can affect thrombus formation at increased shear rates [11]. Worth mentioning is that although P2Y_12_ receptor inhibitors affected the absolute values of platelet adhesion in our study, they did not affect the values of the relative decrease in platelet adhesion after GPIb inhibition, i.e., the GPIb-mediated component of platelet adhesion. Unlike integrins, the GPIb receptor is a mechanosensing receptor. High shear rates, but not platelet activation, trigger the shear-stress-resistant binding of GPIb to VWF [3]. This allows us to evaluate the contribution of GPIb receptors to platelet adhesion without considering the effect of antiplatelet agents.

According to the results of this study, VWF:Ag levels were not associated with premature CAD. In the majority of studies, the association between CAD and VWF was assessed using VWF:Ag levels [12,13,14]. Studies often showed an association between VWF:Ag levels and the risk of major adverse cardiovascular events (MACE) in patients with CAD, while such an association was not observed in healthy subjects [15,16,17]. For example, a prospective ECAT study showed that patients with stable CAD who experienced MI or sudden cardiac death during a 2-year follow-up period initially had higher VWF:Ag levels than patients who did not experience these events. The relative risk of MACE was 85% higher in patients in the upper quantile of VWF:Ag than in patients in the lower quantile [18]. Studies also showed an association between VWF:Ag levels and MACE occurrence in patients with MI [15,16]. In contrast to patients with CAD, VWF:Ag levels are a weak indicator of CAD risk in individuals without this disease [19].

Furthermore, patients without CAD in this study had higher VWF:Ag levels. This could be due to several reasons. VWF:Ag levels can vary over a wide range in the same person at different times [20]. VWF:Ag levels depend on blood group, age, smoking, concomitant diseases, medication intake, and even physical activity [15,21,22].

Moreover, VWF:Ag levels were not associated with platelet adhesion before and after GPIb inhibition in this study. A VWF:Ag level measurement allows for the determination of the total plasma content of VWF but cannot discriminate between its functionally active and inactive forms. Therefore, the functional state of VWF may be a more relevant indicator of a tendency to arterial thrombosis than VWF:Ag levels.

## 5. Conclusions

The VWF-mediated component of platelet adhesion was more pronounced in patients with premature CAD. This may indicate a possible role for excessive VWF–platelet interactions in the development of premature CAD.

## Figures and Tables

**Table 1:** Clinical characteristics of patients.

	Patients with Premature CAD (n = 84)	Control Group (n = 64)	*p*
Age, years	54 (49; 55)	50.5 (44.5; 58)	0.19
Men/women	61 (73%)/ 23 (27%)	26 (42%)/ 38 (58%)	<0.001
Family history of CAD	23 (28%)	10 (16%)	0.11
LDL cholesterol >3 mmol/L	64 (76%)	40 (62%)	0.004
HDL cholesterol <1.0 mmol/L for men and <1.2 mmol/L for women	41 (50%)	14 (25%)	0.004
Smoking Active smoker Former smoker Smoking index Heavy smoker	53 (63%) 33 (62%) 20 (38%) 33 (25; 44) 38 (72%)	24 (38%) 14 (58%) 10 (42%) 17 (8; 22) 5 (21%)	0.003 0.03 0.3 <0.001 <0.001
Obesity	44 (52%)	26 (41%)	0.31
Diabetes mellitus	18 (21%)	5 (8%)	0.03
Arterial hypertension	75 (89%)	52 (81%)	0.23

CAD—coronary artery disease; LDL—low-density lipoprotein cholesterol; HDL—high-density lipoprotein cholesterol; smoking index—the average number of cigarettes smoked per day multiplied by the number of years of smoking and divided by 20; heavy smoker—smoking index ≥25.

**Table 2:** Platelet adhesion in patients with premature CAD and in the control group.

	Platelet Adhesion at Baseline, mV	Platelet Adhesion after GPIb Inhibition, mV	Relative Decrease in Platelet Adhesion after GPIb Inhibition (Δ), %	*p*
Patients with premature CAD (n = 84)	8.3 (5.2; 13.5)	1.7 (1.3; 3.3)	77.0 (57.2; 84.6)	<0.001
Control group (n = 64)	12.6 (9.2; 17.0)	10.3 (4.3; 14.9)	29.6 (0.0; 59.7)	<0.001

CAD—coronary artery disease; mV—millivolt; mAb—monoclonal antibodies; GPIb—glycoprotein Ib; Δ—change in platelet adhesion after blocking platelet GPIb receptor mAb compared to its initial value; *p*—comparison of two dependent variables (Wilcoxon test).

**Table 3:** Univariate logistic regression analysis of the relationship between the probability of premature CAD and selected independent variables.

	Coefficient (β)	OR (95% CI)	*p*
A relative decrease in platelet adhesion after GPIb receptor inhibition, per 1%	0.41	1.04 (1.03–1.06)	<0.001
Male sex	1.36	3.88 (1.94–7.74)	<0.001
Diabetes mellitus	1.17	3.22 (1.13–9.21)	0.029
HDL cholesterol <1.0 mmol/L for men and <1.2 mmol/L for women	1.10	3.00 (1.43–6.31)	0.004
Smoking	1.05	2.85 (1.45– 5.58)	0.002
Age	0.04	1.04 (1.00– 1.08)	0.045
Family history of CAD	0.73	2.07 (0.90– 4.74)	0.085
Arterial hypertension	0.65	1.92 (0.76– 4.89)	0.170
LDL cholesterol >3 mmol/L	0.44	1.55 (0.76–3.18)	0.230
Obesity	0.39	1.48 (0.76–2.88)	0.247

CAD—coronary artery disease; GPIb—glycoprotein Ib; LDL—low-density lipoprotein cholesterol; HDL—high-density lipoprotein cholesterol; OR—odds ratio; CI—confidence interval.

**Table 4:** Multivariate logistic regression analysis of the relationship between the probability of premature CAD and independent variables.

	Coefficient (β)	aOR (95% CI)	*p*
A relative decrease in platelet adhesion after GPIb receptor inhibition, per 1%	0.03	1.03 (1.02–1.05)	<0.001
Male sex	1.09	2.98 (1.13–7.89)	0.03
Age	0.10	1.11 (1.04–1.18)	0.001
Smoking	0.97	2.64 (1.09–6.36)	0.03
Intercept	−5.6	0.004	0.001

GPIb—glycoprotein Ib; aOR—adjusted odds ratio; CI—confidence interval.

## Data Availability

The data used in this article are available upon request without undue reservation.

## Supplementary Materials

The following supporting information can be downloaded at: https://www.mdpi.com/article/10.3390/biomedicines11071916/s1, The logistic regression model used in this study is provided in the Supplementary Materials and includes Tables S1–S4 and Figure S1.
