# Supplementary material for: Platelet Adhesion Mediated by von Willebrand Factor at High Shear Rates Is Associated with Premature Coronary Artery Disease
Source: Biomedicines. 2023 Jul 6;11(7):1916. doi: 10.3390/biomedicines11071916 (PMC10377430; doi:10.3390/biomedicines11071916)
Supplement: Supplementary file 1 [file biomedicines-11-01916-s001.zip › biomedicines-2482957-supplementary.pdf]

Regression analysis

Materials and methods

A probability that a patient has premature coronary artery disease (CAD) was assessed using binary logistic regression. This method calculates a probability of an event (dependent variable) depending on values of independent variables.

A probability of an event is calculated using the following formula:

$$\mathrm{F(z)} = \frac{1}{1+e^{-z}}$$

$Z = b_1 * x_1 + b_2 * x_2 + \ldots + b_n * x_n + C$;
b_1, b_2, b_n – coefficients calculated using binary logistic regression;
x_1, x_2, x_3 – independent variable values;
C– intercept.

Logistic regression analysis was performed for the data of 84 patients with premature CAD verified using coronary angiography and 64 patients without CAD.

The following parameters were taken as probable predictors of premature CAD: sex, age, family history of CAD, diabetes mellitus, hyperlipidemia, arterial hypertension, obesity, smoking, and a relative decrease in platelet adhesion after inhibition of GPIb receptors.

Significant predictors included in the regression model was determined using the Wald test. To assess the quality of the model, we used the Hosmer-Lemeshev, Nagelkerke R^2, and ROC analyses. In addition, the results of the ROC-analysis, as well as the Youden's index were used to calculate the optimal cut-off value.

Regression analysis was performed using IBM SPSS Statistics 26.0, as well as the statistical software package Stata17.

Calculation of regression coefficients. Construction of the prognostic model.

According to the Wald test ($p<0.05$), the following predictors were included in the regression model: male sex, smoking, age and a relative decrease in platelet adhesion after inhibition of GPIb receptors.

Table S1. Variables in the regression equation

Variables in the equation	Coefficient (β)	Standard error	Wald	Significance	Odds ratio	95% confidence interval for odds ratio	
						Lower	Upper
Male sex	1.092	0.497	4.832	0.028	2.981	1.126	7.893
Smoking	0.969	0.45	4.639	0.031	2.635	1.091	6.363
Age	0.101	0.031	10.537	0.001	1.106	1.041	1.176
A relative decrease in platelet adhesion after inhibition of GPIb receptors, per 1%	0.033	0.007	22.722	<0.001	1.033	1.019	1.047
Intercept	-7.786	1.919	16.466	<0.001	<0.001	–	–

Regression equation

$$F(z) = \frac{1}{1+e^{-z}}$$

$$Z = 1.092 * Sex + 0.969 * Smoking + 0.101 * Age + 0.033 * A\ percentage\ decrease\ in\ platelet\ adhesion - 7.786.$$

Evaluation of the resulting model

Table S2. Evaluation of the resulting model

Parameter	Hosmer-Lemeshev goodness of fit	Nagelkerke R^2
Value	0.053	0.479

The Hosmer-Lemeshev test shows that the observed event rate corresponds to the expected event rate in subgroups of the model population.

Nagelkerke R^2 shows that the model is acceptable for use.

ROC analysis

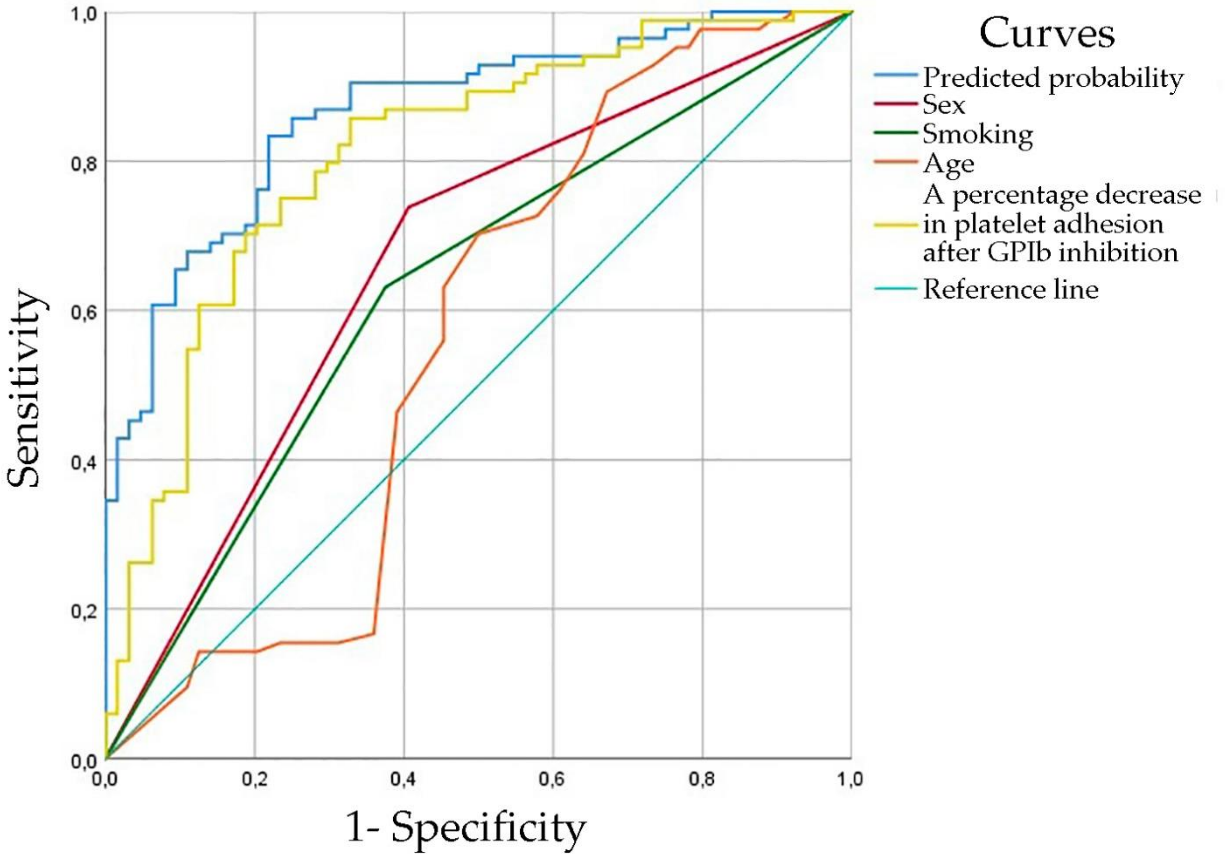

Table S3. Parameters of the ROC analysis

Test result variables: predicted probability					
Risk factors	Area under the curve	Standard error	Asymptotic significance	Asymptotic 95% confidence interval	
				Lower	Upper
Regression model	0.867	0.029	<0.001	0.810	0.924
Sex	0.666	0.046	0.001	0.576	0.755
Smoking	0.628	0.046	0.008	0.537	0.719
Age	0.563	0.051	0.191	0.463	0.663
A relative decrease in platelet adhesion after inhibition of GPIb receptors, per 1%	0.812	0.036	<0.001	0.741	0.883

Table S4. Classification table of the regression model

	Predicted outcome		
Observed outcome	No CAD	CAD	Percentage of correct predictions
No CAD	50	14	78.1%
CAD	14	70	83.3%
Overall percentage of correct predictions			81.1%

At F(z) ≥ 0.586 (cut-off value) CAD is probable (Sensitivity 83.3%, specificity 78.1%).

Conclusion: this model is applicable for determining patients with CAD. Further verification of the model should be performed on more data.
